# Clinical outcomes of chemotherapy in patients with undifferentiated carcinoma of the pancreas: a retrospective multicenter cohort study

**DOI:** 10.1186/s12885-020-07462-4

**Published:** 2020-10-01

**Authors:** Hiroshi Imaoka, Masafumi Ikeda, Kosuke Maehara, Kumiko Umemoto, Masato Ozaka, Satoshi Kobayashi, Takeshi Terashima, Hiroto Inoue, Chihiro Sakaguchi, Kunihiro Tsuji, Kazuhiko Shioji, Keiya Okamura, Yasuyuki Kawamoto, Rei Suzuki, Hirofumi Shirakawa, Hiroaki Nagano, Makoto Ueno, Chigusa Morizane, Junji Furuse

**Affiliations:** 1 Department of Hepatobiliary and Pancreatic Oncology, National Cancer Center Hospital East, 6-5-1, Kashiwanoha, Kashiwa, Chiba, 277-8577 Japan; 2 Department of Hepatobiliary and Pancreatic Oncology, National Cancer Center Hospital, Tokyo, Japan; 3 Department of Clinical Oncology, St.Marianna University School of Medicine, Kawasaki, Japan; 4 Department of Gastroenterological Medicine, Cancer Institute Hospital Japanese Foundation for Cancer Research, Tokyo, Japan; 5 Department of Gastroenterology, Hepatobiliary and Pancreatic Medical Oncology Division, Kanagawa Cancer Center, Yokohama, Japan; 6 Department of Gastroenterology, Kanazawa University Hospital, Kanazawa, Japan; 7 Division of Gastrointestinal Oncology, Shizuoka Cancer Center, Shizuoka, Japan; 8 Department of Gastroenterology, Shikoku Cancer Center, Matsuyama, Japan; 9 Department of Gastroenterology, Ishikawa Prefectural Central Hospital, Kanazawa, Japan; 10 Department of Internal Medicine, Niigata Cancer Center Hospital, Niigata, Japan; 11 Division of Pancreato-Biliary Section, Department of Gastroenterology, JA Sapporo Kohsei Hospital, Sapporo, Japan; 12 Division of Cancer Center, Hokkaido University Hospital, Sapporo, Japan; 13 Department of Gastroenterology, Fukushima Medical University School of Medicine, Fukushima, Japan; 14 Department of Hepato-Biliary-Pancreatic Surgery, Tochigi Cancer Center, Utsunomiya, Japan; 15 Department of Gastroenterological, Breast and Endocrine Surgery, Yamaguchi University Graduate School of Medicine, Ube, Japan; 16 Department of Medical Oncology, Kyorin University Faculty of Medicine, Tokyo, Japan

**Keywords:** Undifferentiated carcinoma, Pancreatic cancer, Anaplastic carcinoma, Chemotherapy, Osteoclast-like giant cells, Paclitaxel, Gemcitabine, Predictor

## Abstract

**Background:**

Undifferentiated carcinoma (UC) of the pancreas is a rare subtype of pancreatic cancer. Although UC has been considered a highly aggressive malignancy, no clinical studies have addressed the efficacy of chemotherapy for unresectable UC. Therefore, we conducted multicenter retrospective study to investigate the efficacy of chemotherapy in patients with UC of the pancreas.

**Methods:**

This multicenter retrospective cohort study was conducted at 17 institutions in Japan between January 2007 and December 2017. A total of 50 patients treated with chemotherapy were analyzed.

**Results:**

The median overall survival (OS) in UC patients treated with chemotherapy was 4.08 months. The details of first-line chemotherapy were as follows: gemcitabine (*n* = 24), S-1 (*n* = 12), gemcitabine plus nab-paclitaxel (*n* = 6), and other treatment (*n* = 8). The median progression-free survival (PFS) was 1.61 months in the gemcitabine group, 2.96 months in the S-1 group, and 4.60 months in the gemcitabine plus nab-paclitaxel group. Gemcitabine plus nab-paclitaxel significantly improved PFS compared with gemcitabine (*p* = 0.014). The objective response rate (ORR) was 4.2% in the gemcitabine group, 0.0% in the S-1 group, and 33.3% in the gemcitabine plus nab-paclitaxel group. Gemcitabine plus nab-paclitaxel also showed a significantly higher ORR compared with both gemcitabine and S-1 (gemcitabine plus nab-paclitaxel vs. gemcitabine: *p* = 0.033; gemcitabine plus nab-paclitaxel vs. S-1: *p* = 0.034). A paclitaxel-containing first-line regimen significantly improved OS compared with a non-paclitaxel-containing regimen (6.94 months vs. 3.75 months, respectively; *p* = 0.041). After adjustment, use of a paclitaxel-containing regimen in any line was still an independent predictor of OS (hazard ratio for OS, 0.221; 95% confidence interval, 0.076–0.647; *p* = 0.006) in multiple imputation by chained equation.

**Conclusions:**

The results of the present study indicate that a paclitaxel-containing regimen would offer relatively longer survival, and it is considered a reasonable option for treating patients with unresectable UC.

## Background

Pancreatic cancer (PC) is one of the deadliest cancers and the fourth leading cause of cancer death in the United States. It has been estimated that, in 2020, approximately 47,050 patients will die of this disease [1]. Global data showed that 448,000 patients were diagnosed with PC in 2017, with a 2.3-times increase in the number of incident cases and deaths from 1990 to 2017 [2]. Despite efforts to develop new treatments [3, 4], the prognosis of PC patients remains poor, and the morbidity is increasing. Undifferentiated carcinoma (UC) of the pancreas, also known as anaplastic carcinoma of the pancreas, is a rare subtype of PC and accounts for 0.3–7% of malignant neoplasms of the pancreas [5–7]. UC is an epithelial neoplasm displaying no particular differentiation such as glandular formation, mucin production, or keratinization.

One population-based study reported that the median age at diagnosis of UC was 67 years, with a slight male predominance (57.5%) [6]. These characteristics are similar to those of PC. On the other hand, UC of the pancreas has been considered more aggressive than PC, and median overall survival (OS) does not exceed 6 months [6, 7]. Clark et al. reported a population-based study comparing patients with UC and PC [6]. The median OS was 11 months in the PC group and 3 months in the UC group, and it was significantly shorter in the UC group than in the PC group (hazard ratio [HR], 1.9; 95% confidence interval [CI], 1.7–2.1). Paal et al. reported 35 patients with UC, and their median OS was 5.2 months [8]. However, these data were based primarily on surgical series or registry data. Considering that it has been reported that approximately 80% of PC patients were diagnosed at unresectable stages [9, 10], the majority of UC cases potentially have metastases. However, previous reports have rarely mentioned unresectable stage disease, and clinical and treatment data for patients with unresectable UC are lacking. Thus, identification of effective chemotherapy regimens for UC is crucial for improving the prognosis of patients with UC.

Therefore, clinical and treatment data of patients with unresectable UC were retrospectively collected. The aim of this multicenter retrospective cohort study was to investigate the efficacy of chemotherapy in patients with UC of the pancreas.

## Methods

### Study design

**Fig. 1:**
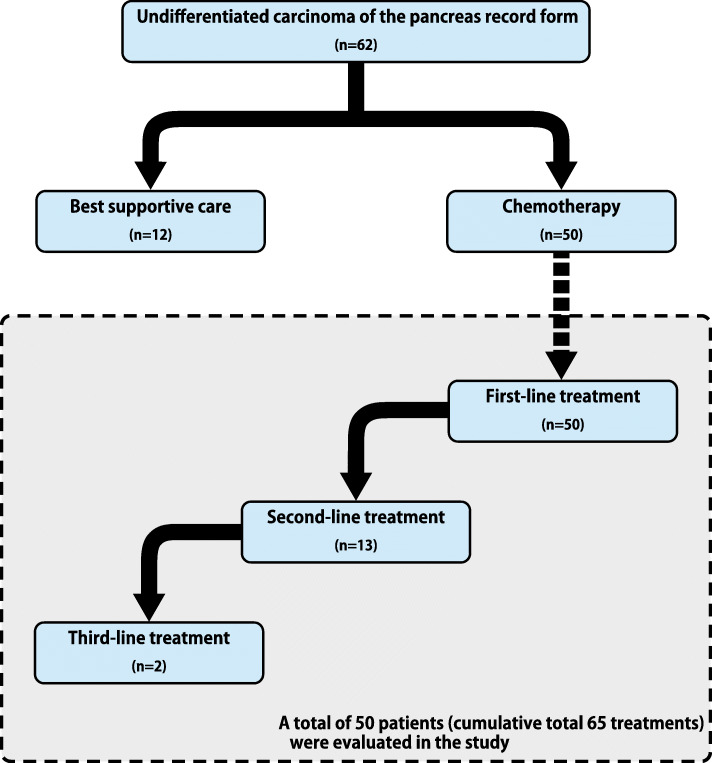
Selection of patients for the study

This retrospective study was conducted at 17 institutions in Japan between January 2007 and December 2017 (Fig. 1). The inclusion criteria were histopathologically diagnosed UC of the pancreas (including UC with osteoclast-like giant cells [UC-OGCs]), recurrent/metastatic or locally advanced disease, and treated with chemotherapy. The study protocol was approved by the institutional review boards of the participating institutions.

### Patient evaluation

Data regarding clinical and laboratory features, histological findings, treatment, and outcome measures were collected retrospectively. Histological findings were based on pathology reports and classified into the following subtypes: anaplastic type, sarcomatoid type, carcinosarcoma, UC-OGCs, and not otherwise specified (NOS) [11].

OS was measured from the date of start of first-line treatment to the date of death from any cause. OS for patients who were lost to follow-up was censored at the last date they were known to be alive. PFS was measured from the date of start of treatment to the date of first documented disease progression or the date of death from any cause. PFS was censored at the time of the last follow-up if there was no documentation of disease progression or death. Tumor response was based on the best overall response throughout the entire course of the observation period. The Response Evaluation Criteria in Solid Tumors (RECIST) version 1.1 were used to assess tumor responses [12].

### Statistical analysis

Univariate analysis was performed using the chi-squared test for categorical variables. The Kaplan-Meier method was used to estimate the time-to-event distribution, and *p*-values were calculated using the log-rank test. HRs were calculated using the Cox proportional hazards model. Values of *p* < 0.05 were considered statistically significant, and all p-values are two-sided. To analyze predictors of OS in patients with unresectable UC, a multivariate Cox proportional hazards model was used, including predictors (*p* < 0.10) on univariate analysis and clinically relevant variables (Eastern Cooperative Oncology Group performance status [ECOG PS], extent of disease, and histological subtype). Due to the retrospective analysis, covariate data were often missing. Thus, multiple imputation was performed by multiple imputation by chained equation (MICE) to avoid potential bias [13]. A Cox proportional hazards model was performed in complete-case analysis and MICE. Data were analyzed using STATA version 15.1 (StataCorp, College Station, TX, USA) and R version 3.6.1 (http://www.r-project.org/).

## Results

### Patient characteristics

A cumulative total of 65 treatments were given to 50 UC patients between January 2007 and December 2017. The baseline characteristics of the patients with UC are shown in Table 1. ECOG PS was 0 in 13 patients (26.0%), 1 in 31 patients (62.0%), and ≥ 2 in 4 patients (8.0%). The median treatment line was 1 (range 1–3). A total of 13 patients received second-line treatment, and 2 patients received third-line treatment. The details of chemotherapy in any treatment line were as follows: gemcitabine (*n* = 27), S-1 (*n* = 18), gemcitabine plus nab-paclitaxel (*n* = 9), FOLFIRINOX (*n* = 4), gemcitabine plus S-1 (n = 2), paclitaxel (n = 1), and other treatment (n = 4).

**Table 1:** Patient baseline characteristics

	All patients	
	(*n* = 50)	Missing
Sex		
Male (%)	34 (68.0)	
Female (%)	16 (32.0)	
Age (y)		
Median (range)	69 (41–83)	
ECOG PS		
0 (%)	13 (26.0)	2 (4.0)
1 (%)	31 (62.0)	
≥ 2 (%)	4 (8.0)	
Prior surgical resection	25 (50.0)	
Tumor location		
Head (%)	24 (48.0)	
Body-Tail (%)	26 (52.0)	
Tumor size (cm)		
Median (range)	4.5 (2.0–18.0)	1 (2.0)
Extent of disease		
Locally advanced (%)	6 (12.0)	
Metastatic (%)	44 (88.0)	
Measurable metastatic sites		
Liver (%)	26 (52.0)	
Lymph node (%)	20 (40.0)	
Lung (%)	4 (8.0)	
Peritoneal (%)	12 (24.0)	
LDH, U/L		
Median (range)	205 (128–909)	2 (4.0)
CRP, mg/L		
Median (range)	13 (0–178)	2 (4.0)
CEA, ng/mL		
Median (range)	3.0 (0.7–64.1)	1 (2.0)
CA19–9, U/mL		
Median (range)	35.7 (1.0–43,645)	1 (2.0)
Histological subtype		
Anaplastic type (%)	16 (32.0)	
Sarcomatoid type (%)	4 (8.0)	
Undifferentiated carcinoma with OGCs (%)	11 (22.0)	
NOS (%)	19 (38.0)	

*ECOG PS* Eastern Cooperative Oncology Group performance status, *LDH* lactate dehydrogenase, *CRP* C-reactive protein, *CEA* carcinoembryonic antigen, *CA19–9* carbohydrate antigen 19–9, *OGCs* osteoclast-like giant cells, *NOS* not otherwise specified

### Overall treatment efficacy

**Fig. 2:**
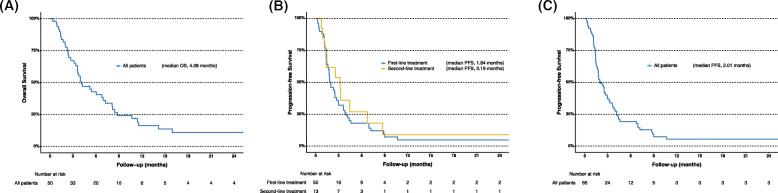
Kaplan-Meier curves of overall survival for all patients with undifferentiated carcinoma of the pancreas (**a**), progression-free survival with first-line and second-line treatments (**b**), and progression-free survival for the cumulative total of 65 treatments (**c**). OS, overall survival; PFS, progression-free survival

**Fig. 3:**
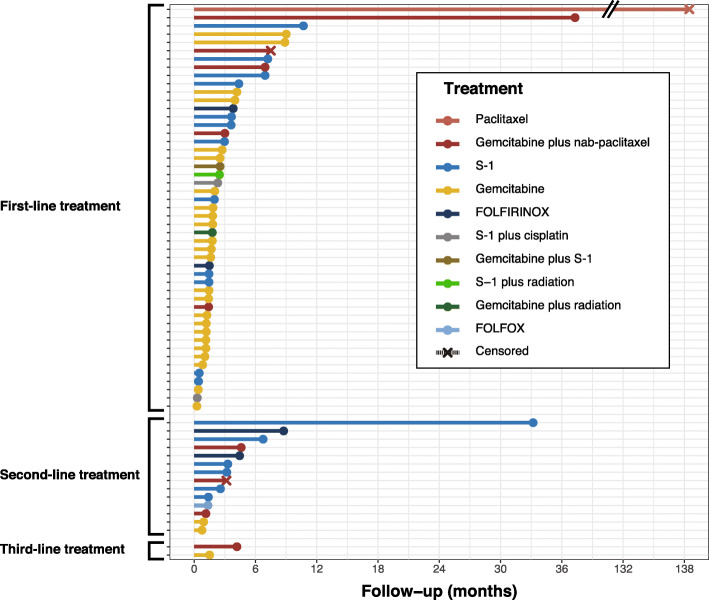
Individual progression-free survival of patients treated with chemotherapy

For all patients treated with chemotherapy, the median OS was 4.08 months, and the 12-month OS rate was 16.3% (Fig. 2a). The median PFS in patients receiving first-line treatment and in those receiving second-line treatment was 1.84 months and 3.19 months, respectively (Fig. 2b). For the cumulative total of 65 treatments, the median PFS was 2.01 months (Fig. 2c). The individual PFS for each is shown in Fig. 3, and their objective response rate (ORR) was 10.8%.

### Efficacy of first-line treatment

**Fig. 4:**
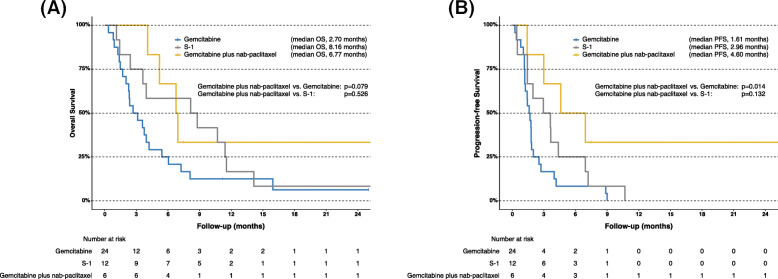
Kaplan-Meier curves of overall survival in the first-line gemcitabine group, S-1 group, and gemcitabine plus nab-paclitaxel group (**a**); and progression-free survival in the first-line gemcitabine group, S-1 group, and gemcitabine plus nab-paclitaxel group (**b**). OS, overall survival; PFS, progression-free survival

The details of chemotherapy in first-line treatment were as follows: gemcitabine (*n* = 24), S-1 (*n* = 12), gemcitabine plus nab-paclitaxel (*n* = 6), and other treatment (*n* = 8). The median OS in the first-line gemcitabine group, S-1 group, and gemcitabine plus nab-paclitaxel group was 2.70 months, 8.16 months, and 6.77 months, respectively (Fig. 4a). The median PFS in the first-line gemcitabine group, S-1 group, and gemcitabine plus nab-paclitaxel group was 1.61 months, 2.96 months, and 4.60 months, respectively (Fig. 4b). Tumor responses in first-line treatment are shown in Table 2. There was no significant difference in OS among UC patients treated with gemcitabine, S-1, and gemcitabine plus nab-paclitaxel. However, gemcitabine plus nab-paclitaxel significantly improved PFS compared with gemcitabine (*p* = 0.014), and it showed significantly higher ORR compared with both gemcitabine and S-1 (gemcitabine plus nab-paclitaxel vs. gemcitabine: *p* = 0.033; gemcitabine plus nab-paclitaxel vs. S-1: *p* = 0.034).

**Table 2:** Tumor response in each line of treatment

	Total number	CR	PR	SD	PD	NE	ORR	DCR
**1st-line treatment**								
**All patients**	**50**	**1**	**4**	**12**	**27**	**6**	**10.0%**	**34.0%**
Gemcitabine	24	0	1	5	15	3	4.2%	25.0%
S-1	12	0	0	5	6	1	0.0%	41.7%
Gemcitabine plus nab-paclitaxel	6	0	2	1	2	1	33.3%	50.0%
Gemcitabine plus S-1	2	0	0	0	2	0		
FOLFIRINOX	2	0	1	0	1	0		
S-1 plus radiation	1	0	0	1	0	0		
Paclitaxel	1	1	0	0	0	0		
S-1 plus cisplatin	1	0	0	0	1	0		
Gemcitabine plus radiation	1	0	0	0	0	1		
**2nd-line treatment**								
**All patients**	**13**	**0**	**1**	**4**	**4**	**4**	**7.7%**	**38.5%**
S-1	6	0	0	3	1	2	0.0%	50.0%
Gemcitabine	2	0	0	0	1	1		
Gemcitabine plus nab-paclitaxel	2	0	0	0	1	1		
FOLFIRINOX	2	0	1	1	0	0		
FOLFOX	1	0	0	0	1	0		
**3rd-line treatment**								
**All patients**	2	0	1	1	0	0		
Gemcitabine	1	0	0	1	0	0		
Gemcitabine plus nab-paclitaxel	1	0	1	0	0	0		
**Cumulative total**								
**All patients**	**65**	**1**	**6**	**17**	**31**	**10**	**10.8%**	**36.9%**
Gemcitabine	27	0	1	6	16	4	3.7%	25.9%
S-1	18	0	0	8	7	3	0.0%	44.4%
Gemcitabine plus nab-paclitaxel	9	0	3	1	3	2	33.3%	44.4%

*CR* complete response, *PR* partial response, *SD* stable disease, *PD* progressive disease, *NE* not evaluable, *PFS* progression-free survival, *CI* confidence interval, *NR* not reached

### Efficacy of paclitaxel-containing regimens

**Fig. 5:**
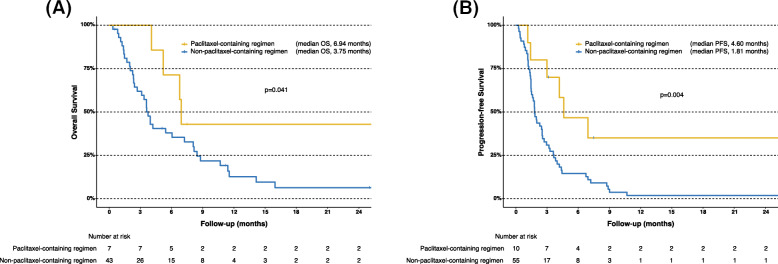
Kaplan-Meier curves comparing overall survival in patients treated with a first-line paclitaxel-containing regimen and a non-paclitaxel-containing regimen (**a**), and comparing progression-free survival in patients treated with a paclitaxel-containing regimen and a non-paclitaxel-containing regimen in any line (**b**). OS, overall survival; PFS, progression-free survival

Two different paclitaxel-containing regimens (gemcitabine plus nab-paclitaxel (*n* = 6) and paclitaxel monotherapy (*n* = 1)) were used as first-line treatment. A paclitaxel-containing first-line regimen significantly improved OS compared with a non-paclitaxel-containing regimen (6.94 months vs. 3.75 months, respectively; *p* = 0.041) (Fig. 5a). For the cumulative total of 65 treatments, a paclitaxel-containing regimen significantly improved PFS compared with a non-paclitaxel-containing regimen (4.60 months vs. 1.81 months, respectively; *p* = 0.004) (Fig. 5b). ORR was significantly higher with a paclitaxel-containing regimen than with a non-paclitaxel-containing regimen (40.0% vs. 5.5%, respectively; *p* = 0.001). In addition, one complete response was observed in a patient treated with paclitaxel monotherapy, and this patient achieved long survival.

The results of the Cox proportional hazards model for predicting OS in patients treated with chemotherapy are shown in Table 3. In the univariate Cox proportional hazards model, use of a paclitaxel-containing regimen in any line and absence of liver metastasis were significant factors associated with OS. In the multivariate Cox proportional hazards model, use of a paclitaxel-containing regimen in any line was still an independent predictor of OS (HR for OS, 0.221; 95% CI, 0.076–0.647; *p* = 0.006) in MICE.

**Table 3:** Predictors of survival in patients treated with chemotherapy

	Complete-case analysis (*n* = 44)						Multiple imputation by chained equation (*n* = 50)					
	Unadjusted HR			Adjusted HR			Unadjusted HR			Adjusted HR		
	Estimate	95% CI	*P*	Estimate	95% CI	*P*	Estimate	95% CI	*P*	Estimate	95% CI	*P*
Sex												
Male (Reference)	1.000						1.000					
Female	0.537	0.255 — 1.131	0.102				0.606	0.303 — 1.213	0.157			
Age, y												
< 65 (Reference)	1.000			1.000			1.000			1.000		
≥ 65	1.982	0.981 — 4.004	0.057	3.404	1.472 — 7.875	0.004	1.877	0.981 — 3.591	0.057	2.242	1.119 — 4.494	0.023
ECOG PS												
0 (Reference)	1.000			1.000			1.000			1.000		
≥ 1	1.384	0.626 — 3.060	0.422	1.709	0.752 — 3.885	0.201	1.292	0.638 — 2.619	0.477	1.293	0.627 — 2.667	0.486
Prior surgical resection												
No (Reference)	1.000						1.000					
Yes	0.798	0.414 — 1.537	0.500				0.760	0.413 — 1.397	0.377			
Tumor location												
Head (Reference)	1.000						1.000					
Body-Tail	1.124	0.574 — 2.200	0.733				1.153	0.619 — 2.145	0.654			
Extent of disease												
Locally advanced (Reference)	1.000			1.000			1.000			1.000		
Metastatic	0.820	0.316 — 2.132	0.685	0.630	0.210 — 1.891	0.410	0.820	0.319 — 2.107	0.681	0.606	0.204 — 1.805	0.369
Location of metastases												
Liver	2.173	1.111 — 4.248	0.023	2.373	1.086 — 5.184	0.030	1.919	1.034 — 3.559	0.039	1.721	0.856 — 3.457	0.127
Lymph node	0.805	0.413 — 1.570	0.525				0.831	0.442 — 1.563	0.566			
Peritoneal	0.865	0.406 — 1.842	0.707				0.933	0.458 — 1.901	0.849			
LDH, U/L												
≤ 250 (Reference)	1.000						1.000					
> 250	0.715	0.324 — 1.576	0.405				0.829	0.407 — 1.686	0.604			
CRP, mg/L												
≤ 10 (Reference)	1.000						1.000					
> 10	1.624	0.846 — 3.116	0.145				1.650	0.898 — 3.031	0.106			
CEA, ng/mL												
≤ 5.0 (Reference)	1.000						1.000					
> 5.0	1.239	0.600 — 2.559	0.562				1.337	0.675 — 2.650	0.405			
CA19–9, U/mL												
≤ 37.0 (Reference)	1.000						1.000					
> 37.0	1.032	0.537 — 1.981	0.926				1.143	0.620 — 2.107	0.669			
Histological subtype												
UC without OGCs (Reference)	1.000			1.000			1.000			1.000		
UC with OGCs	0.935	0.385 — 2.268	0.882	2.372	0.848 — 6.635	0.100	0.857	0.378 — 1.943	0.713	0.826	0.354 — 1.930	0.660
Use of paclitaxel-containing regimen in any line												
No (Reference)	1.000			1.000			1.000			1.000		
Yes	0.216	0.076 — 0.620	0.004	0.181	0.062 — 0.534	0.002	0.218	0.077 — 0.621	0.004	0.221	0.076 — 0.647	0.006

*HR* hazard ratio, *CI* confidence interval, *ECOG PS* Eastern Cooperative Oncology Group performance status, *LDH* lactate dehydrogenase, *CRP* C-reactive protein, *CEA* carcinoembryonic antigen, *CA19–9* carbohydrate antigen 19–9, *UC* undifferentiated carcinoma, *OGCs* osteoclast-like giant cells

## Discussion

Using a retrospective cohort design, the present study examined the efficacy of chemotherapy in patients with UC of the pancreas. The most frequently used first-line treatment regimens were gemcitabine, S-1, and gemcitabine plus nab-paclitaxel. Although there was no significant difference in OS among these first-line regimens, gemcitabine plus nab-paclitaxel significantly improved PFS compared with gemcitabine, and it showed a significantly higher ORR compared with both gemcitabine and S-1. In addition, one complete response was observed in a patient treated with paclitaxel. A paclitaxel-containing first-line regimen significantly improved OS compared with a non-paclitaxel-containing regimen. After adjustment, use of a paclitaxel-containing regimen in any line was still an independent predictor of OS. All these observations indicate that a paclitaxel-containing regimen is a reasonable option for treatment of patients with unresectable UC of the pancreas.

The present study showed that most UC patients were diagnosed at the age of 60–70 years, with a slight male predominance. These clinical features were similar to those of PC [2], but median OS for UC patients treated with chemotherapy did not exceed 5 months. In recent phase 3 trials for metastatic PC [14, 15], the median OS reached approximately 1 year. The present study clearly showed that UC was refractory to chemotherapy. Of the chemotherapeutic regimens used for UC, gemcitabine monotherapy was the most frequently used regimen for unresectable UC in the present study; it provided a limited response, with median PFS and an ORR of 1.61 months and 3.7%, respectively. Most patients with unresectable UC had lower ECOG PS at the time of diagnosis, and the majority of patients received only one line of treatment. Although gemcitabine monotherapy still remains a therapeutic option for frail and elderly patients with PC [16–18], the benefit of gemcitabine monotherapy may be limited for UC patients. On the other hand, paclitaxel-containing regimens may have a relatively high anti-tumor effect in UC. Paclitaxel has shown activity in anaplastic carcinoma of the thyroid [19, 20] and sarcoma (e.g. angiosarcoma and Kaposi’s sarcoma) [21–24]. It has been reported that UC of the pancreas expressed epithelial-mesenchymal transition (EMT) markers (e.g. Slug, Twist, Zeb1), as in anaplastic carcinoma of the thyroid and sarcoma [8, 25]. Drug sensitivity of UC of the pancreas may be similar to these neoplasms because they show similar pathological features and expression of EMT markers.

EMT is a complex process by which epithelial cells lose their cell polarity and cell-cell adhesion, and they gain migratory and invasive properties to mesenchymal cells. Accumulating evidence indicates that EMT plays a crucial role in cancer-related events, including cancer invasion and metastasis. EMT is known to be associated with a poor prognosis in various cancers, and this fact may contribute to the aggressive clinical course of UC. On the other hand, the mechanism of the effect of paclitaxel on UC is unclear. Paclitaxel, including nab-paclitaxel, is a widely used chemotherapy drug for various cancers, including PC [26–28]. However, many studies have reported that the EMT is associated with acquired resistance to chemotherapy drugs [29–31], including paclitaxel [32]. The EMT is known as a heterogeneous phenomenon and the progression of cancer varies depending on the EMT phenotype [33]. Mattiolo et al. reported that the EMT was also expressed in UC, both with and without OGCs [23]. Ishida et al. categorized UC into 2 subgroups based on the expression patterns of EMT markers and E-cadherin. They suggested that these differences in EMT phenotypes may have an impact on the prognosis of UC [24]. Given these findings, the EMT status may have an impact on the response to paclitaxel in UC. However, further elucidation is required to understand differences in drug responses.

In addition to the efficacy of paclitaxel-containing regimens, a multivariate Cox proportional hazard model showed that age ≥ 65 years was an independent predictor of OS in patients treated with chemotherapy. Fundamentally, chemotherapy provides a modest survival benefit in patients with unresectable UC. However, some patients had a good response to chemotherapy and achieved relatively long survival. In such a situation, there is a critical need to identify high-risk patients and select patients who will potentially benefit from treatment based on predictors. For example, for patients who are not expected to respond to chemotherapy, it is possible to avoid highly invasive treatments and focus on quality of life. By predicting the chemotherapeutic response, it makes a significant contribution to the selection of treatment for UC. Age is a widely accepted prognostic factor for PC, and Clark et al. reported that age was an independent prognostic factor for survival in UC (HR per 10 years, 1.1; 95% CI, 1.04–1.2) in their population-based study [6]. This report supports the present findings. It should be noted that the survival benefit of chemotherapy for UC may be limited in patients aged ≥65 years.

Future directions in research on UC will lead to identification of biomarkers for therapeutic stratification, such as microsatellite instability-high in various types of cancers [34] and *EGFR* in lung cancer [35]. Currently, there is no established treatment specific to UC. Thus, most UC patients were treated by chemotherapy in accordance with PC, but the result of the present study showed limited survival benefit of chemotherapy. Presently, precision medicine, tailoring treatment based on an individual’s genetics, lifestyle, and environment, has emerged. Development of technologies can provide a comprehensive view of an individual patient’s cancer [36, 37], which can impact real-time clinical decision-making. For UC patients, precision medicine has not been well established. However, newer technologies [38] can unveil various potential predictive biomarkers for possible development of new treatments and precision medicine.

This study has some limitations. The first limitation is the fact that the present analysis was a retrospective study that lacked adequate statistical power due to the small sample size. Therefore, the study should be considered only an exploratory investigation. However, UC of the pancreas is a rare malignant neoplasm, and this could complicate the recruiting for and completion of clinical trials for UC of the pancreas. The retrospective design and relatively small sample size limit the strength of this study, and the effects of chemotherapy need to be evaluated in a larger patient cohort. However, the result is not negligible because the results will benefit patients with UC for whom it has been difficult to establish therapeutic strategies. The second limitation is missing values. Due to the retrospective nature of this study, missing data were unavoidable, which may lead to bias and loss of information in the study [39]. It may undermine the value of such a small-sized study for a rare disease. Thus, multiple imputation was used to account for missing values. The prognostic factors obtained by multiple imputation may be useful in decision-making for the treatment of UC of the pancreas. The final limitation is the small number of patients treated with FOLFIRINOX. Of the 4 patients treated with FOLFIRINOX, two had partial response. FOLFIRINOX may be potentially effective for UC of the pancreas. However, even so, UC patients are often in poor condition. Thus, less invasive treatment than FOLFIRINOX, gemcitabine plus nab-paclitaxel, is a reasonable choice for UC of the pancreas [16].

## Conclusions

The results of the present retrospective multicenter cohort study show that paclitaxel-containing regimens would offer relatively longer survival, and they are considered a reasonable option for treating patients with unresectable UC.

## Data Availability

The datasets used and/or analyzed during the current study are available from the corresponding author on reasonable request.

## Abbreviations

CI: Confidence interval
ECOG PS: Eastern Cooperative Oncology Group performance status
HR: Hazard ratio
MICE: Multiple imputation by chained equation
NOS: Not otherwise specified
ORR: Objective response rate
OS: Overall survival
PC: Pancreatic cancer
PFS: Progression-free survival
RECIST: Response Evaluation Criteria in Solid Tumors
UC: Undifferentiated carcinoma
UC-OGCs: Undifferentiated carcinoma with osteoclast-like giant cells
